# Tumor-infiltrating lymphocytes and breast cancer mortality in racially and ethnically diverse participants of the Northern California Breast Cancer Family Registry

**DOI:** 10.1093/jncics/pkae023

**Published:** 2024-03-28

**Authors:** Julia D Ransohoff, Iain Miller, Jocelyn Koo, Vishal Joshi, Allison W Kurian, Kimberly H Allison, Esther M John, Melinda L Telli

**Affiliations:** Department of Medicine (Oncology), Stanford University School of Medicine, Palo Alto, CA, USA; Stanford Cancer Institute, Stanford University School of Medicine, Palo Alto, CA, USA; Department of Pathology, Stanford University School of Medicine, Palo Alto, CA, USA; Stanford Cancer Institute, Stanford University School of Medicine, Palo Alto, CA, USA; Department of Medicine (Oncology), Stanford University School of Medicine, Palo Alto, CA, USA; Department of Medicine (Oncology), Stanford University School of Medicine, Palo Alto, CA, USA; Stanford Cancer Institute, Stanford University School of Medicine, Palo Alto, CA, USA; Department of Epidemiology and Population Health, Stanford University School of Medicine, Palo Alto, CA, USA; Stanford Cancer Institute, Stanford University School of Medicine, Palo Alto, CA, USA; Department of Pathology, Stanford University School of Medicine, Palo Alto, CA, USA; Department of Medicine (Oncology), Stanford University School of Medicine, Palo Alto, CA, USA; Stanford Cancer Institute, Stanford University School of Medicine, Palo Alto, CA, USA; Department of Epidemiology and Population Health, Stanford University School of Medicine, Palo Alto, CA, USA; Department of Medicine (Oncology), Stanford University School of Medicine, Palo Alto, CA, USA; Stanford Cancer Institute, Stanford University School of Medicine, Palo Alto, CA, USA; Department of Epidemiology and Population Health, Stanford University School of Medicine, Palo Alto, CA, USA

## Abstract

Stromal tumor-infiltrating lymphocyte (sTIL) enrichment in pretreatment breast tumors has been associated with superior response to neoadjuvant treatment and survival. In a population-based cohort, we studied sTIL-survival associations by race and ethnicity. We assessed associations of continuous sTIL scores and sTIL-enriched breast cancers (defined as percent lymphocytic infiltration of tumor stroma or cell nests at cutoffs of 30%, 50%, and 70%) with clinical and epidemiologic characteristics and conducted multivariable survival analyses. Although we identified no difference in sTIL score by race and ethnicity, higher continuous sTIL score was associated with lower breast cancer–specific mortality only among non-Hispanic White and Asian American but not African American and Hispanic women. This finding suggests that complex factors influence treatment response and survival, given that sTIL enrichment was not associated with a survival advantage among women from minoritized groups, who more often experience health disparities. Further study of patient selection for sTIL-guided treatment strategies is warranted.

Stromal tumor-infiltrating lymphocyte (sTIL) enrichment in pretreatment breast cancer specimens predicts better response to neoadjuvant chemotherapy (1), improved disease-free and overall survival in early-stage triple-negative breast cancer (TNBC) treated with chemotherapy (2,3), and higher pathologic complete response rate and disease-free survival in HER2-positive disease (4). More recent data suggest an association between sTILs and response to checkpoint inhibitor monotherapy in metastatic TNBC (5) and to combination neoadjuvant chemoimmunotherapy (6). Identifying characteristics associated with sTILs and survival may inform therapeutic escalation or de-escalation strategies. Prior sTIL studies focused primarily on non-Hispanic White (NHW) patients and demonstrated geographic variation in sTIL scores (4,7), suggesting that differences in race and ethnicity, genetics, or exposures may drive antitumor immunity.

We assessed sTIL associations with clinical, epidemiologic, and genetic characteristics and survival outcomes in the Northern California Breast Cancer Family Registry (NC-BCFR) (8,9). We hypothesized that sTIL-survival associations vary by race and ethnicity.

## Methods

The study was based on women diagnosed at younger than age 65 years with a first primary invasive breast cancer ascertained through the population-based Greater Bay Area Cancer Registry. Cases diagnosed younger than age 35 years, with a family history of breast cancer, and from minoritized populations were oversampled per the NC-BCFR sampling scheme, as previously described (9). All research was approved by Institutional Review Boards of the Cancer Prevention Institute of California and Stanford University. All participants provided informed consent at study enrollment. H&E slides were available for cases diagnosed between 1995 and 2005. TILs were independently scored in deciles (1,2,10) by 2 pathologists, and averaged scores were calculated as the percentage of stromal area containing mononuclear infiltrates; intraepithelial TILs (iTILs) were calculated as the percentage of lymphocytes in contact with tumor cells. An sTIL score of 0% was coded as 0%; all other scores were rounded up to the next highest decile. The sTIL scores differing by at least 20% were adjudicated. The sTIL enrichment was defined as 30% or greater, 50% or greater, or 70% or greater sTILs (2).

Tumor characteristics (age at diagnosis, stage, histology, grade, nodal involvement, size, ER/PR/HER2 status) were obtained from the California Cancer Registry (CCR). Patient characteristics (race and ethnicity, education, marital status, breast cancer family history, parity, time since last full-term pregnancy, menopausal status, weight, height) were collected by enrollment interview. Germline *BRCA1*/2 pathogenic variant (PV) testing was completed in NC-BCFR (11). Vital status and cause of death were obtained from NC-BCFR follow-up through April 30, 2022 or CCR linkage through 2018.

Interrater TIL scoring reliability was assessed by weighted kappas and 95% confidence intervals (CIs). Differences in sTIL scores by tumor and patient characteristics were determined using analysis of variance and in sTIL enrichment status using the χ^2^ test. Multivariable Cox proportional hazards models were fit to estimate hazard ratios (HRs) and 95% confidence intervals for breast cancer–specific mortality (BCM) and overall mortality (OM). We confirmed that the proportional hazards assumption was met. Patients alive through the observation period were censored at the last follow-up date. Time to OM and BCM were compared within the sTIL-enriched (≥50%) and nonenriched (<50%) subgroups using Kaplan-Meier survival estimates. For BCM, patients were censored at the date of non-breast-cancer-related death. To test for heterogeneity in hazard ratios by race and ethnicity, we included in the Cox model an interaction term of each exposure variable (per decile sTIL increase, sTIL enrichment status) with race and ethnicity. Two-sided *P* value less than .05 was considered statistically significant. Analyses were performed using SAS 9.4 (SAS Institute, Inc., Cary NC).

## Results

Among 355 samples, sTILs were not assessed in a subset because they contained only in situ carcinoma (n = 31), lymph node tissue (n = 2), or poor stain quality (n = 38). Patients diagnosed with stage IV disease (n = 5) were excluded from survival analyses. Of the remaining 279 cases, 64% were African American, Asian American, or Hispanic; 99.3% and 98.2% of patients were tested for PV in *BRCA1* or *BRCA2*, respectively (Supplementary Table 1, available online). Follow-up ranged from 1.3 to 26.6 years (mean = 15.3, median = 18.0).

Interrater reliability was high for sTILs (kappa = 0.81, 95% CI = 0.78 to 0.84) and lower for iTILs (0.37, 95% CI = 0.25 to 0.49). Mean sTIL score was higher than mean iTIL score (26.2 vs 1.9, *P* < .01). The sTILs at or greater than 50% were observed in 16.9% (47/279) of cases, 4% (5/125) of hormone receptor-positive (ER/PR-positive, HER2-negative) cases, 23.0% (14/61) of HER2-positive cases, and 33.8% (24/71) of TNBC cases (Supplementary Table 1, available online). The sTILs at or greater than 50% were present in 16.8% (17/101) of NHW cases, 21.9% (16/73) of Asian American cases, 8.1% (3/37) of African American cases, and 16.4% (11/67) of Hispanic cases. The distribution of clinicopathologic characteristics at sTIL enrichment cutoffs of 30% or greater, 50% or greater, and 70% or greater is detailed in Supplementary Table 1 (available online). The sTIL and iTIL distributions in the study sample, including representative H&E slide images, and by patient and tumor characteristics are shown in the Supplement (Supplementary Tables 1–3, Supplementary Figure 1, available online).

In multivariable analyses, higher sTIL score (per decile increase) was associated with lower BCM in the overall cohort (HR = 0.85, 95% CI = 0.74 to 0.99) (Table 1). The association with BCM held for hormone receptor-negative (ER/PR-negative) disease (per decile increase: 0.76, 95% CI = 0.62 to 0.92) and suggestively for TNBC (per decile increase: 0.78, 95% CI = 0.60 to 1.03).

**Table 1.** Multivariable Cox proportional hazards regression models of associations of sTILs with breast cancer–specific and overall mortality

		Breast cancer-specific mortality					Overall mortality				
Group	Cases	Deaths	Hazard ratio (95% CI)				Deaths	Hazard ratio (95% CI)			
	N	N					N				
			sTIL	sTILs	sTILs	sTILs		sTIL	sTILs	sTILs	sTILs
			Per decile increase	≥30% vs <30%	≥50% vs <50%	≥70% vs <70%		Per decile increase	≥30% vs <30%	≥50% vs <50%	≥70% vs <70%
Overall^a^	279	66	0.85 (0.74 to 0.99)	0.53 (0.30 to 0.94)	0.53 (0.24 to 1.18)	0.61 (0.19 to 1.98)	95	0.92 (0.83 to 1.03)	0.72 (0.46 to 1.14)	0.75 (0.41 to 1.37)	1.08 (0.49 to 2.37)
Breast cancer subtype^a^											
ER^+^ and/or PR^+^	156	36	0.94 (0.71 to 1.23)	0.56 (0.19 to 1.64)	0.45 (0.06 to 3.44)	—^d^	51	1.01 (0.81 to 1.25)	0.91 (0.41 to 2.01)	0.77 (0.18 to 3.32)	0.95 (0.13 to 7.18)
ER^-^/PR^-^	123	30	0.76 (0.62 to 0.92)	0.34 (0.16 to 0.75)	0.39 (0.15 to 1.02)	0.73 (0.21 to 2.49)	44	0.84 (0.72 to 0.98)	0.49 (0.26 to 0.91)	0.61 (0.29 to 1.27)	1.11 (0.45 to 2.75)
TNBC	71	15	0.78 (0.60 to 1.03)	0.32 (0.10 to 1.00)	0.33 (0.08 to 1.30)	1.00 (0.26 to 3.87)	23	0.92 (0.75 to 1.13)	0.51 (0.20 to 1.26)	0.92 (0.35 to 2.47)	0.92 (0.35 to 2.47)
Race and ethnicity^b^^,^^c^											
African American	37	11	1.05 (0.72 to 1.53)	0.84 (0.21 to 3.32)	3.23 (0.49 to 21.3)	—^e^	14	0.99 (0.70 to 1.38)	0.58 (0.07 to 4.65)	2.34 (0.42 to 13.1)	0.99 (0.94 to 1.05)
Asian American	73	14	0.59 (0.37 to 0.96)	0.14 (0.03 to 0.73)	0.25 (0.03 to 2.03)	0.63 (0.08 to 5.21)	16	0.63 (0.41 to 0.95)	0.20 (0.05 to 0.79)	0.20 (0.03 to 1.59)	0.58 (0.07 to 4.65)
Hispanic	67	13	0.94 (0.66 to 1.32)	0.43 (0.11 to 1.70)	1.23 (0.29 to 5.15)	3.01 (0.35 to 25.7)	23	0.94 (0.72 to 1.24)	0.59 (0.22 to 1.63)	1.06 (0.33 to 3.44)	1.68 (0.21 to 13.3)
Non-Hispanic White	101	28	0.74 (0.58 to 0.94)	0.38 (0.15 to 0.96)	0.12 (0.02 to 0.91)	0.28 (0.04 to 2.11)	42	0.88 (0.74 to 1.04)	0.60 (0.30 to 1.21)	0.48 (0.19 to 1.25)	0.87 (0.30 to 2.49)
*P* for interaction by race and ethnicity	–	–	0.16	0.26	0.04	—		0.22	0.28	0.07	0.41

a Models for breast cancer–specific and overall mortality for the overall cohort and for breast cancer subtypes were adjusted for prognostic variables including age at diagnosis (continuous), race and ethnicity (non-Hispanic White, Asian American, African American, Hispanic), stage (stage I, stage II, stage III, unknown), prediagnosis BMI (<25, 25-29.9, ≥30 kg/m^2^), and germline *BRCA2* pathogenic variant status (positive, negative, not tested). CI = confidence interval; sTIL = stromal tumor infiltrating lymphocyte; ER^+^ = estrogen receptor-positive; ER^−^ = estrogen receptor-negative; PR^+^ = progesterone receptor-positive; PR^-^ = progesterone receptor-negative; TNBC = triple-negative breast cancer (ER^-^, PR^-^, HER2^-^).

b Models for breast cancer-specific and overall mortality by race and ethnicity were adjusted for age at diagnosis (continuous), stage (stage I, stage II, III, unknown), prediagnosis BMI (<30, ≥30 kg/m^2^), and germline *BRCA2* pathogenic variant status (positive, negative, not tested).

c Excludes 1 Native American patient.

d No breast cancer deaths among patients with sTILs at or greater than 70%.

e No patients with sTILs at or greater than 70%.

**Figure 1.**
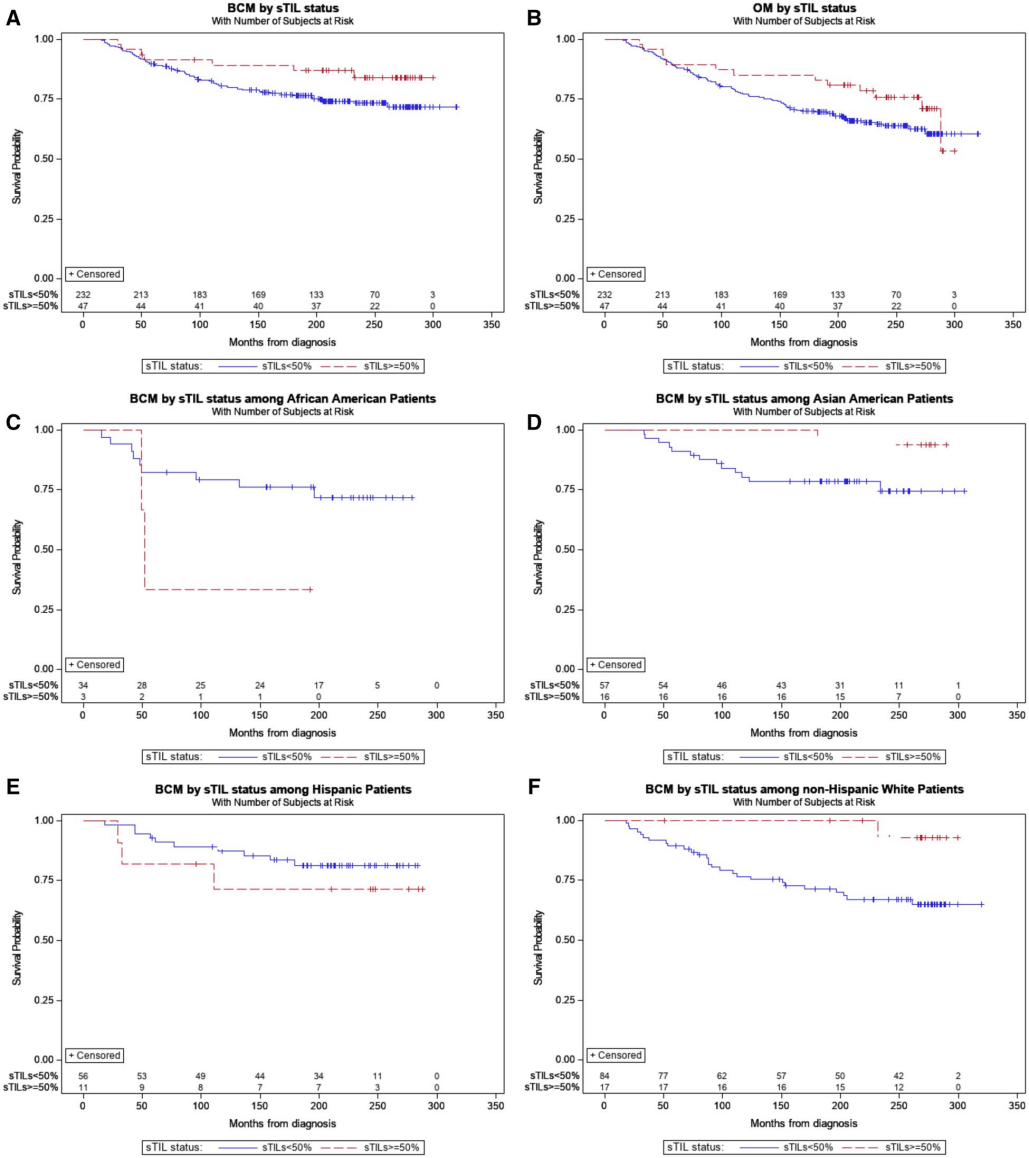
Kaplan-Meier estimates of breast cancer–specific and overall mortality by stromal TIL (sTIL) enrichment status overall (A-B) and breast cancer-specific mortality by racial and ethnic subgroups (C-F). Inset numbers represent the number of patients at risk at each point in time. BCM = breast cancer–specific mortality; OM = overall mortality.

In the overall cohort, higher sTIL score (per decile increase) was associated with lower BCM among NHW (0.74, 95% CI = 0.58 to 0.94) and Asian American (0.59, 95% CI = 0.37 to 0.96) cases and lower OM among Asian American (0.63, 95% CI = 0.41 to 0.95) and suggestively among NHW (0.88, 95% CI = 0.74 to 1.04) cases. The sTILs at or greater than 50% were associated with lower BCM among NHW cases (0.12, 95% CI = 0.02 to 0.91) (Figure 1) but not Asian American cases (0.25, 95% CI = 0.03 to 2.03) and were not associated with OM in either group. By contrast, among African American and Hispanic cases, higher sTIL score (per decile increase) was not associated with BCM or OM. At an sTIL enrichment cutoff of at or greater than 50%, no associations were seen with BCM or OM among African American or Hispanic cases. There was a statistically significant interaction between sTILs at or greater than 50% and BCM by race and ethnicity (*P*_interaction_ = .04 for sTILs ≥50%) and a borderline significant interaction for OM by race and ethnicity (*P*_interaction_ = .07 for sTILs ≥50%).

In the subset of patients with ER/PR-negative disease, we found a significant interaction between sTILs at or greater than 50% and OM by race and ethnicity (*P*_interaction_ = .04 for sTILs ≥50%).

## Discussion

We identified a novel sTIL-survival association that varied by race and ethnicity. In contrast to recent work that found more sTILs in breast tumors of Black than non-Black patients (12,13) and lower OM in Black patients with sTIL-enriched tumors (14), we found no difference in sTIL score by racial and ethnic group, and sTIL enrichment was not associated with better survival among African American and Hispanic women. Although prior work also showed that TILs in breast cancers of Black women displayed an exhausted T-cell phenotype, this signature was prognostic only in ER/PR-positive tumors in which sTILs were not associated with survival (14).

The current findings, in the context of related retrospective trial analyses, suggest that sTIL phenotype—particularly in ER/PR-negative and sTIL-enriched tumors—is related to survival, and the predictive and prognostic value of sTILs may vary by race and ethnicity. This finding is particularly timely given the 2021 incorporation of immunotherapy into early-stage TNBC treatment (15) and ongoing trials of neoadjuvant treatment de-escalation with chemotherapy omission for TIL-enriched ER/PR-negative tumors [BELLINI (16) and the upcoming EORTC 2257 BCG/OPTImaL]. The current findings suggest TIL enrichment may not confer an equivalent survival advantage for all women, warranting caution with patient selection for TIL-guided treatment approaches, and underscoring the pressing need for comprehensively exploring existing trial and population-based data in pooled analyses, as well as through prospective investigations. The impact of immunotherapy on potentially mitigating TIL-associated survival disparities—and particularly whether Black patients may derive a greater benefit from immunotherapy^13^—will be a critical area of future study. Potential mechanisms for less TIL benefit among African American and Hispanic women, who have inferior breast cancer survival (17-19) and are under-represented in breast cancer research, include genetic differences in TIL biology, delays in treatment initiation (20,21), treatment and treatment adherence differences (22), and physiologic dysregulation secondary to allostatic load stressors related to structural inequity and systemic inequality (23).

Study limitations include the relatively small numbers of participants in subgroups of interest, which limited statistical power. Only first-course treatment information before the immunotherapy era (15) was available from the CCR, and recurrences are not recorded in the CCR. However, given the long follow-up period, BCM represents sequelae of recurrence. Strengths include the multiethnic population-based sample with a high proportion of sTIL-enriched tumors, near-complete germline *BRCA1/2* testing, a relatively young cohort (63% diagnosed at age <50 years, mean = 45.5) enriched for the aggressive tumor subtypes seen among younger women and reflective of the NC-BCFR sampling scheme (9), and nearly 20-year follow-up.

This work raises key questions for future studies, including how immune biomarkers such as TILs should be used to inform interventional trial design of treatment de-escalation or escalation approaches—particularly in African American and Hispanic women—and how immunotherapy may overcome survival disparities. The findings have direct potential to inform future prospective investigations of TIL biology and TIL-guided treatment approaches.

## Supplementary Material

pkae023_Supplementary_Data

## Data Availability

The data are available from Dr Esther M. John upon reasonable request.
