# Supplementary material for: Tumor-infiltrating lymphocytes and breast cancer mortality in racially and ethnically diverse participants of the Northern California Breast Cancer Family Registry
Source: JNCI Cancer Spectr. 2024 Mar 28;8(2):pkae023. doi: 10.1093/jncics/pkae023 (PMC11031224; doi:10.1093/jncics/pkae023)

**Supplementary Tables and Figures**

Supplementary Table 1. Baseline Tumor and Patient Characteristics Overall and by Stromal Tumor-infiltrating Lymphocyte Status.

| **Characteristic** | **Cases** | **sTIL Score** | **p-value**^a^ | **sTILs ≥30%** | **sTILs <30%** | **p-value^b^** | **sTILs ≥50%** | **sTILs <50%** | **p-value**^b^ | **sTILs ≥70%** | **sTILs <70%** | **p-value**^b^ |
| --- | --- | --- | --- | --- | --- | --- | --- | --- | --- | --- | --- | --- |
|  | N (%)^c^ | Mean (95% CI) |  | N (%)^d^ | N (%)^d^ |  | N (%)^d^ | N (%)^d^ |  | N (%)^d^ | N (%)^d^ |  |
|  | 279 (100) | 26.6 (24.3 - 28.9) |  | 99 (35) | 180 (65) |  | 47 (17) | 232 (83) |  | 17 (6) | 262 (94) |  |
| **AJCC stage** |  |  | <0.01 |  |  | 0.24 |  |  | 0.63 |  |  | 0.59 |
| I | 109 (41) | 22.6 (19.0 - 26.2) |  | 30 (28) | 79 (72) |  | 14 (13) | 95 (87) |  | 5 (5) | 104 (95) |  |
| II | 137 (51) | 30.8 (27.6 - 34.0) |  | 62 (45) | 75 (55) |  | 30 (22) | 107 (78) |  | 10 (7) | 127 (93) |  |
| III | 21 (8) | 21.0 (12.8 - 29.1) |  | 4 (19) | 17 (81) |  | 1 (5) | 20 (95) |  | 1 (5) | 20 (95) |  |
|  |  |  |  |  |  |  |  |  |  |  |  |  |
| **Histology** |  |  | 0.03 |  |  | 0.25^e,f^ |  |  | 0.47^e,f^ |  |  | 0.25^e,f^ |
| Ductal | 239 (87) | 27.3 (24.8 - 29.7) |  | 89 (37) | 150 (63) |  | 43 (18) | 196 (82) |  | 13 (5) | 226 (95) |  |
| Lobular | 14 (5) | 13.6 (3.4 - 23.8) |  | 1 (7) | 13 (93) |  | 0 (0) | 14 (100) |  | 0 (0) | 14 (100) |  |
| Other | 21 (8) | 29.5 (21.2 - 37.8) |  | 8 (38) | 13 (62) |  | 4 (19) | 17 (81) |  | 4 (19) | 17 (81) |  |
|  |  |  |  |  |  |  |  |  |  |  |  |  |
| **Grade** |  |  | <0.01 |  |  | <0.01 |  |  | <0.01 |  |  | 0.04 |
| Low | 25 (10) | 14.2 (7.3 - 21.1) |  | 2 (8) | 23 (92) |  | 1 (4) | 24 (96) |  | 0 (0) | 25 (100) |  |
| Intermediate | 100 (38) | 19.2 (15.8 - 22.6) |  | 18 (18) | 82 (82) |  | 5 (5) | 95 (95) |  | 4 (4) | 96 (96) |  |
| High | 136 (52) | 35.5 (32.6 - 38.5) |  | 77 (57) | 59 (43) |  | 40 (29) | 96 (71) |  | 12 (9) | 124 (91) |  |
|  |  |  |  |  |  |  |  |  |  |  |  |  |
| **Nodal status** |  |  | 0.08 |  |  | 0.08 |  |  | 0.52 |  |  | 0.07 |
| Negative | 153 (59) | 24.8 (21.7 - 27.9) |  | 48 (31) | 105 (69) |  | 24 (16) | 129 (84) |  | 6 (4) | 147 (96) |  |
| Positive | 107 (41) | 29.1 (25.4 - 32.7) |  | 45 (42) | 62 (58) |  | 20 (19) | 87 (81) |  | 10 (9) | 97 (91) |  |
|  |  |  |  |  |  |  |  |  |  |  |  |  |
| **Tumor size (T-stage)^g^** |  |  | 0.10 |  |  | 0.51 |  |  | 0.89 |  |  | 0.15 |
| T1 | 156 (60) | 25.4 (22.3 - 28.4) |  | 50 (32) | 106 (68) |  | 25 (16) | 131 (84) |  | 12 (8) | 144 (92) |  |
| T2 | 92 (35) | 30.4 (26.5 - 34.4) |  | 43 (47) | 49 (53) |  | 19 (21) | 73 (79) |  | 4 (4) | 88 (96) |  |
| T3 | 14 (5) | 22.9 (12.7 - 33.0) |  | 3 (21) | 11 (79) |  | 1 (7) | 13 (93) |  | 0 (0) | 14 (100) |  |
|  |  |  |  |  |  |  |  |  |  |  |  |  |
| **Breast cancer subtype** |  |  | <0.01 |  |  | <0.01 |  |  | <0.01 |  |  | <0.01 |
| ER/PR^+^/HER2^-^ | 125 (45) | 17.5 (14.5 - 20.6) |  | 19 (15) | 106 (85) |  | 5 (4) | 120 (96) |  | 2 (2) | 123 (98) |  |
| TNBC | 71 (25) | 38.0 (33.9 - 42.0) |  | 44 (62) | 27 (38) |  | 24 (34) | 47 (66) |  | 11 (15) | 60 (85) |  |
| ER/PR^-^/HER2^+^ | 30 (11) | 34.8 (28.6 - 41.1) |  | 16 (53) | 14 (47) |  | 10 (33) | 20 (67) |  | 2 (7) | 28 (93) |  |
| ER/PR^+^/HER2^+^ | 31 (11) | 25.5 (19.3 - 31.6) |  | 10 (32) | 21 (68) |  | 4 (13) | 27 (87) |  | 1 (3) | 30 (97) |  |
| ER/PR^-^/HER2 unknown | 22 (8) | 31.6 (24.3 - 38.9) |  |  |  |  | 4 (18) | 18 (82) |  | 1 (5) | 21 (95) |  |
|  |  |  |  |  |  |  |  |  |  |  |  |  |
| ***BRCA1* PV status** |  |  | 0.07 |  |  | 0.10^f^ |  |  | 0.56^f^ |  |  | 0.13^f^ |
| Negative | 256 (92) | 26.0 (23.6 - 28.3) |  | 87 (34) | 169 (66) |  | 42 (16) | 214 (84) |  | 14 (5) | 242 (95) |  |
| Positive | 21 (8) | 34.0 (25.7 - 42.4) |  | 11 (52) | 10 (48) |  | 5 (24) | 16 (76) |  | 3 (14) | 18 (86) |  |
|  |  |  |  |  |  |  |  |  |  |  |  |  |
| ***BRCA2* PV status** |  |  | 0.86 |  |  | 0.75^f^ |  |  | 0.88^f^ |  |  | 0.51^f^ |
| Negative | 263 (96) | 26.7 (24.3 - 29.0) |  | 95 (36) | 168 (64) |  | 45 (17) | 218 (83) |  | 16 (6) | 247 (94) |  |
| Positive | 11 (4) | 27.7 (16.1 - 39.3) |  | 3 (27) | 8 (73) |  | 2 (18) | 9 (82) |  | 1 (9) | 10 (91) |  |
|  |  |  |  |  |  |  |  |  |  |  |  |  |
| **Age at diagnosis (years)** |  |  | 0.03 |  |  | 0.05 |  |  | 0.02 |  |  | 0.05 |
| 22-34 | 58 (21) | 32.5 (27.5 - 37.5) |  | 26 (45) | 32 (55) |  | 16 (28) | 42 (72) |  | 7 (12) | 51 (88) |  |
| 35-49 | 116 (42) | 25.9 (22.4 - 29.4) |  | 42 (36) | 74 (64) |  | 18 (16) | 98 (84) |  | 6 (5) | 110 (95) |  |
| 50-64 | 105 (38) | 24.1 (20.4 - 27.7) |  | 31 (30) | 74 (70) |  | 13 (12) | 92 (88) |  | 4 (4) | 101 (96) |  |
|  |  |  |  |  |  |  |  |  |  |  |  |  |
| **Race and ethnicity^h^** |  |  | 0.56 |  |  | 0.63 |  |  | 0.34 |  |  | 0.80 |
| African American | 37 (13) | 23.9 (17.6 - 30.2) |  | 11 (30) | 26 (70) |  | 3 (8) | 34 (92) |  | 0 (0) | 37 (100) |  |
| Asian American | 73 (26) | 29.1 (24.6 - 33.6) |  | 30 (41) | 43 (59) |  | 16 (22) | 57 (78) |  | 7 (10) | 66 (90) |  |
| Hispanic | 67 (24) | 25.7 (21.0 - 30.3) |  | 22 (33) | 45 (67) |  | 11 (16) | 56 (84) |  | 2 (3) | 65 (97) |  |
| Non-Hispanic White | 101 (36) | 26.3 (22.5 - 30.1) |  | 36 (36) | 65 (64) |  | 17 (17) | 84 (83) |  | 8 (8) | 93 (92) |  |
|  |  |  |  |  |  |  |  |  |  |  |  |  |
| **Education level** |  |  | 0.66 |  |  | 0.99 |  |  | 0.15 |  |  | 0.51 |
| Some high school or less | 29 (10) | 27.9 (20.8 - 35.0) |  | 12 (41) | 17 (59) |  | 4 (14) | 25 (86) |  | 2 (7) | 27 (93) |  |
| High school graduate | 49 (18) | 24.5 (19.0 - 30.0) |  | 16 (33) | 33 (67) |  | 6 (12) | 43 (88) |  | 2 (4) | 47 (96) |  |
| Some college/ technical school | 88 (32) | 25.5 (21.4 - 29.5) |  | 29 (33) | 59 (67) |  | 13 (15) | 75 (85) |  | 4 (5) | 84 (95) |  |
| College or higher degree | 113 (41) | 28.0 (24.4 - 31.6) |  | 42 (37) | 71 (63) |  | 24 (21) | 89 (79) |  | 9 (8) | 104 (92) |  |
|  |  |  |  |  |  |  |  |  |  |  |  |  |
| **Marital status** |  |  | 0.17 |  |  | 0.18 |  |  | 0.16 |  |  | 0.13 |
| Married | 183 (66) | 24.4 (20.5 - 28.3) |  | 70 (38) | 113 (62) |  | 35 (19) | 148 (81) |  | 14 (8) | 169 (92) |  |
| Not married | 96 (34) | 27.7 (24.9 - 30.6) |  | 29 (30) | 67 (70) |  | 12 (13) | 84 (88) |  | 3 (3) | 93 (97) |  |
|  |  |  |  |  |  |  |  |  |  |  |  |  |
| **History of breast cancer in 1^st^ degree relative** |  |  | 0.71 |  |  | 0.42 |  |  | 0.03 |  |  | 0.49 |
| No | 175 (63) | 26.9 (24.0 - 29.8) |  | 59 (34) | 116 (66) |  | 36 (21) | 139 (79) |  | 12 (7) | 163 (93) |  |
| Yes | 104 (37) | 26.0 (22.2 - 29.8) |  | 40 (38) | 64 (62) |  | 11 (11) | 93 (89) |  | 5 (5) | 99 (95) |  |
|  |  |  |  |  |  |  |  |  |  |  |  |  |
| **Number of full-term pregnancies** |  |  | 0.72^i^ |  |  | 0.83^i^ |  |  | 0.14^i^ |  |  | 0.10^i^ |
| Nulliparous | 64 (23) | 28.5 (23.7 - 33.3) |  | 24 (38) | 40 (63) |  | 15 (23) | 49 (77) |  |  |  |  |
| 1 | 50 (18) | 24.7 (19.3 - 30.1) |  | 15 (30) | 35 (70) |  | 7 (14) | 43 (86) |  | 6 (9) | 58 (91) |  |
| 2 | 100 (36) | 27.0 (23.2 - 30.8) |  | 39 (39) | 61 (61) |  | 17 (17) | 83 (83) |  | 4 (8) | 46 (92) |  |
| ≥3 | 65 (23) | 25.5 (20.7 - 30.2) |  | 21 (32) | 44 (68) |  | 8 (12) | 57 (88) |  | 5 (5) | 95 (95) |  |
|  |  |  |  |  |  |  |  |  |  | 2 (3) | 63 (97) |  |
| **Years since last full-term pregnancy** |  |  | 0.37^j^ |  |  | 0.29^j^ |  |  | 0.08^j^ |  |  | 0.03^j^ |
| ≥20 | 86 (40) | 24.0 (20.1 - 28.0) |  | 27 (31) | 59 (69) |  | 9 (10) | 77 (90) |  |  |  |  |
| 10-19 | 66 (31) | 25.4 (20.9 - 29.9) |  | 23 (35) | 43 (65) |  | 10 (15) | 56 (85) |  | 3 (3) | 83 (97) |  |
| 4-9 | 41 (19) | 29.8 (24.0 - 35.5) |  | 16 (39) | 25 (61) |  | 8 (20) | 33 (80) |  | 1 (2) | 65 (98) |  |
| ≤3 | 22 (10) | 28.6 (20.8 - 36.4) |  | 9 (41) | 13 (59) |  | 5 (23) | 17 (77) |  | 4 (10) | 37 (90) |  |
|  |  |  |  |  |  |  |  |  |  | 3 (14) | 19 (86) |  |
| **Menopausal status** |  |  | 0.16 |  |  | 0.16 |  |  | 0.34 |  |  | 0.76 |
| Pre-menopausal | 159 (59) | 27.9 (24.9 - 30.9) |  | 62 (39) | 97 (61) |  | 30 (19) | 129 (81) |  | 10 (6) | 149 (94) |  |
| Post-menopausal | 111 (41) | 24.5 (20.9 - 28.1) |  | 34 (31) | 77 (69) |  | 16 (14) | 95 (86) |  | 6 (5) | 105 (95) |  |
|  |  |  |  |  |  |  |  |  |  |  |  |  |
| **BMI (kg/m^2^)** |  |  | 0.05 |  |  | 0.52 |  |  | <0.01 |  |  | 0.10 |
| <25 | 152 (55) | 29.0 (25.9 - 32.1) |  | 60 (39) | 92 (61) |  | 36 (24) | 116 (76) |  | 12 (8) | 140 (92) |  |
| 25-29.9 | 67 (24) | 25.6 (21.0 - 30.2) |  | 24 (36) | 43 (64) |  | 7 (10) | 60 (90) |  | 4 (6) | 63 (94) |  |
| ≥30 | 58 (21) | 21.9 (16.9 - 26.9) |  | 15 (26) | 43 (74) |  | 4 (7) | 54 (93) |  | 1 (2) | 57 (98) |  |

Abbreviations. AJCC: American Joint Committee on Cancer; BMI: body mass index; ER: estrogen receptor; HER2: human epidermal growth factor receptor 2; HR: hormone receptor; LPBC: lymphocyte-predominant breast cancer; PR: progesterone receptor; PV: pathogenic variant; sTIL: stromal tumor-infiltrating lymphocyte; TNBC: triple-negative breast cancer.

ER/PR^+^ represents estrogen receptor-positive or progesterone receptor-positive disease. ER/PR^-^ represents estrogen receptor-negative and progesterone receptor-negative disease. Variants of uncertain significance were categorized as negative for *BRCA1* or *BRCA2* PV status.

^a^ p-value for difference in means from one-way ANOVA.

^b^ Chi square unless otherwise indicated.

^c^ Column percent.

^d^ Row percent.

^e^ Comparison of ductal versus lobular or other histology.

^f^ Fisher’s exact test.

^g^ Tumor size was categorized as per the AJCC 8^th^ edition TNM anatomic stage groups, with T1 representing tumor ≤2cm, T2 >2 to ≤5cm, and T3 >5cm.

^h^ Excludes 1 Native American patient.

^i^ Includes nulliparous women.

^j^ Among parous women only.

Supplementary Table 2. sTIL and iTIL Distributions.

|  | **Breast Cancers with sTILs**  (N = 279) | | **Breast Cancers with iTILs**  (N = 276) | |
| --- | --- | --- | --- | --- |
| **Score** | N | % | **Score** | N |
| 0 | 1 | 0.4 | 0 | 1 |
| 10 | 88 | 32.0 | 10 | 88 |
| 20 | 69 | 24.6 | 20 | 69 |
| 30 | 41 | 14.4 | 30 | 41 |
| 40 | 21 | 7.4 | 40 | 21 |
| 50 | 22 | 8.1 | 50 | 22 |
| 60 | 14 | 4.9 | 60 | 14 |
| 70 | 14 | 4.9 | 70 | 14 |
| 80 | 8 | 2.8 | 80 | 8 |
| 90 | 1 | 0.4 | 90 | 1 |

Abbreviations. sTIL: stromal tumor-infiltrating lymphocyte; iTIL: intraepithelial tumor-infiltrating lymphocyte.

Supplementary Table 3. Association of Mean Stromal TIL Score and Overall and Breast Cancer-Specific Mortality with Age at Diagnosis, Body Mass Index, and Tumor Grade.

|  | **All** | | **Non-Hispanic White** | | **African American** | | **Hispanic** | | **Asian American** | |
| --- | --- | --- | --- | --- | --- | --- | --- | --- | --- | --- |
|  | **N** | **Mean (95% CI)** | **N** | **Mean (95% CI)** | **N** | **Mean (95% CI)** | **N** | **Mean (95% CI)** | **N** | **Mean (95% CI)** |
|  | 279 |  | 101 |  | 37 |  | 67 |  | 73 |  |
|  |  |  |  |  |  |  |  |  |  |  |
| **Age at diagnosis (years)** | p | 0.09 |  | 0.36 |  | 0.61 |  | 0.33 |  | 0.27 |
| <50 | 174 | 28.2 (25.3-31.1) | 63 | 27.9 (22.9-32.9) | 21 | 25.0 (18.7-31.3) | 47 | 27.0 (22.0-32.0) | 42 | 31.5 (24.9-38.1) |
| ≥50 | 105 | 24.1 (20.3-27.8) | 38 | 24.1 (17.7-30.5) | 16 | 22.5 (15.3-29.7) | 20 | 22.5 (14.8-30.2) | 31 | 25.8 (18.1-33.5) |
|  |  |  |  |  |  |  |  |  |  |  |
| **BMI (kg/m^2^)** | p | 0.03 |  | 0.06 |  | 0.86 |  | 0.50 |  | 0.66 |
| <25 | 152 | 29.0 (25.9-32.1) | 67 | 29.8 (24.8-34.7) | 8 | 23.1 (12.9-33.4) | 26 | 27.5 (20.8-34.2) | 56 | 29.7 (24.0-35.5) |
| ≥25 | 125 | 24.0 (20.6-27.4) | 37 | 21.9 (15.5-28.4) | 29 | 24.1 (18.8-29.5) | 41 | 24.5 (19.1-29.9) | 17 | 27.1 (16.6-37.5) |
|  |  |  |  |  |  |  |  |  |  |  |
| **Grade** | p | <0.01 |  | <0.01 |  | <0.01 |  | <0.01 |  | <0.01 |
| Low-intermediate | 125 | 18.3 (15.2-21.4) | 42 | 16.0 (10.8-21.1) | 13 | 15.4 (8.0-22.8) | 36 | 18.8 (13.6-23.9) | 33 | 21.8 (14.7-28.9) |
| High | 136 | 35.5 (32.5-38.5) | 49 | 37.3 (32.6-42.1) | 21 | 29.8 (23.9-35.6) | 28 | 35.4 (29.5-41.2) | 38 | 36.4 (29.8-43.1) |

Abbreviations. BMI: body mass index; CI: confidence interval. P-value is for difference in mean stromal TIL score by characteristic by one-way ANOVA.

Supplementary Figure 1: Variable degrees of intratumoral and stromal lymphocytic infiltration (iTIL, sTIL) on hematoxylin and eosin (HE)–stained tumor slides at 40x magnification. Shown are examples of tumor and associated stroma with no iTILs or sTILs (A); with increased iTILs, with lymphocytes shown in direct contact with tumor cells (B); with 30% sTILs (C); and with 90% sTILs, with abundant lymphocytes seen in the tumor stroma (D).


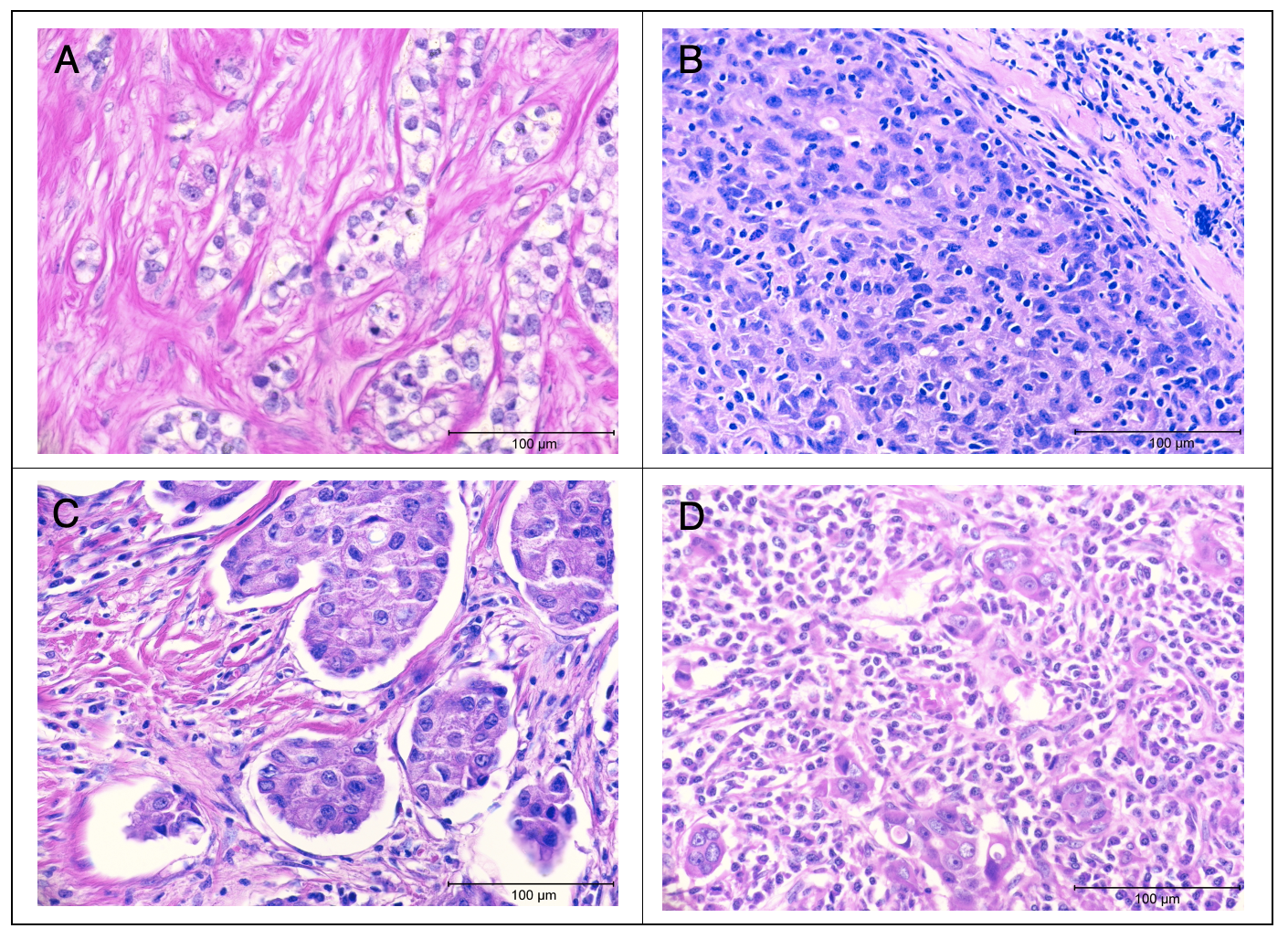

Supplement: pkae023_Supplementary_Data [file pkae023_supplementary_data.docx]
